# Suspected Pulmonary Embolism-Associated Advanced Heart Block

**DOI:** 10.7759/cureus.96756

**Published:** 2025-11-13

**Authors:** Priya Ramcharan, Arun R Katwaroo, Stefon Ramkissoon, Stephanie Harrypersadsingh, Racquel Charles, Valmiki K Seecheran, Rajeev V Seecheran, Naveen A Seecheran

**Affiliations:** 1 Cardiology, Eric Williams Medical Sciences Complex, Mount Hope, TTO; 2 Internal Medicine, St. James Medical Complex, St. James, TTO; 3 Internal Medicine, Eric Williams Medical Sciences Complex, Mount Hope, TTO; 4 Neprhology, University of New Mexico, Albuquerque, USA

**Keywords:** advanced heart block, complete heart block (chb), coronary slow flow (csf), direct oral anticoagulant (doac), pulmonary embolism (pe)

## Abstract

We describe a 35-year-old Caribbean-Black male smoker with suspected pulmonary embolism (PE)-associated advanced heart block, which was both confirmed with computed tomography pulmonary angiography (CTPA) and inpatient telemetry. The patient received guideline-directed medical therapy for PE, including direct oral anticoagulation (DOAC), and underwent permanent dual-chamber pacemaker (DCPM) implantation for complete heart block (CHB). The clinician should consider atypical etiologies such as PE in young adults presenting with advanced heart block.

## Introduction

Complete heart block (CHB), also known as third-degree atrioventricular block (3°AVB), is a severe cardiovascular condition characterized by the complete dissociation of atrial and ventricular impulses, leading to an atrial rate greater than the ventricular rate. Common etiologies include fibrosis of the conduction system, ischemic heart disease, increased vagal tone, and certain medications such as digitalis, calcium channel blockers, beta-blockers, quinine, and amiodarone [1-4].

Pulmonary embolism (PE), a potentially lethal condition, represents the third leading cause of cardiovascular mortality following acute coronary syndromes (ACS) and cerebrovascular events (CVEs). The clinical presentation is diverse, ranging from asymptomatic cases to obstructive shock resulting in death. Electrocardiographic (ECG) findings often provide valuable clues in a PE diagnosis, particularly when clinical suspicion is high. Among the most frequently observed ECG abnormalities in PE are right bundle branch block (RBBB) and the characteristic S1Q3T3 pattern. RBBB reflects a conduction delay in the right ventricle, potentially a consequence of increased right ventricular strain due to PE. The S1Q3T3 pattern, also known as the McGinn-White sign, is a more specific ECG finding for PE, characterized by a deep S wave in lead I, a Q wave in lead III, and T-wave inversion in lead III. The presence of these ECG abnormalities, however, is not pathognomonic for PE and can be observed in other conditions [5-8].

This report documents a case of coincident CHB and PE, a phenomenon rarely reported in the literature [9-12]. We describe a 35-year-old Caribbean-Black male smoker with suspected PE-associated advanced heart block, which were both confirmed with computed tomography pulmonary angiography (CTPA) and inpatient telemetry. The patient received guideline-directed medical therapy (GDMT) for PE, including direct oral anticoagulation (DOAC), and underwent permanent dual-chamber pacemaker (DCPM) implantation for CHB. The clinician should consider atypical etiologies such as PE in young adults presenting with advanced heart block.

## Case presentation

A 35-year-old Caribbean-Black male smoker with no prior medical history presented to the emergency room with presyncope for the previous three days. He described profound lightheadedness and faintness with intermittent atypical angina and dyspnea; however, he did not report any overt syncope or palpitations. His social history was positive for tobacco use for approximately 10 years (10 pack-years of smoking) without any illicit drug use, such as cocaine or narcotic opiates. He did not report any significant family history of premature cardiac disease, recent antecedent infection, or pet or travel history. He also denied any ingestion of any anabolic supplements or complementary and alternative medications.

**Figure 1:**
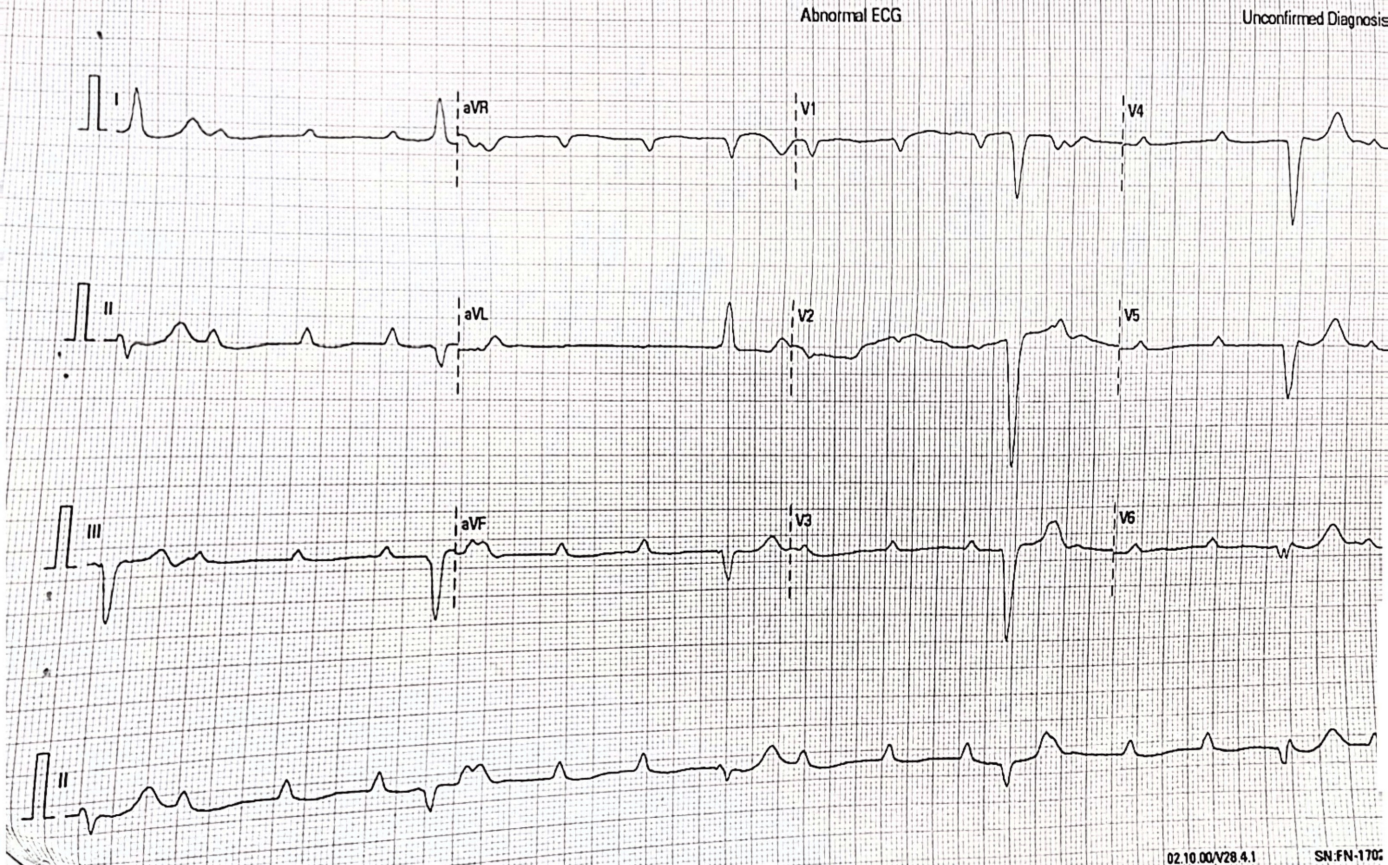
The patient’s 12-lead electrocardiogram (ECG) on presentation to the emergency room Indicated a complete heart block (CHB) with an intraventricular conduction delay (IVCD).

In the emergency room, his vital signs were: elevated blood pressure at 160/72 millimeters of mercury (mmHg; reference range between 90/60 and 120/80 mmHg), bradycardic heart rate at 28 beats per minute (reference range between 60 and 100 bpm), tachypneic respiratory rate at 20 breaths per minute (reference range between 12 and 18 breaths per minute), and oximetry of 98% on room air (reference range between 98 and 100%). Further examination revealed a Glasgow Coma Scale (GCS) score of 15/15, indicating intact neurological function. A cardiopulmonary exam revealed soft S1 and S2 without murmurs, an abnormal jugular venous waveform with suspected canon “a” waves and vesicular breath sounds bilaterally. An abdominal examination revealed mild epigastric tenderness, with regular bowel sounds. The patient’s emergent 12-lead electrocardiogram (ECG) revealed 3°AVB with an intraventricular conduction delay (143 milliseconds) (Figure 1).

**Figure 2:**
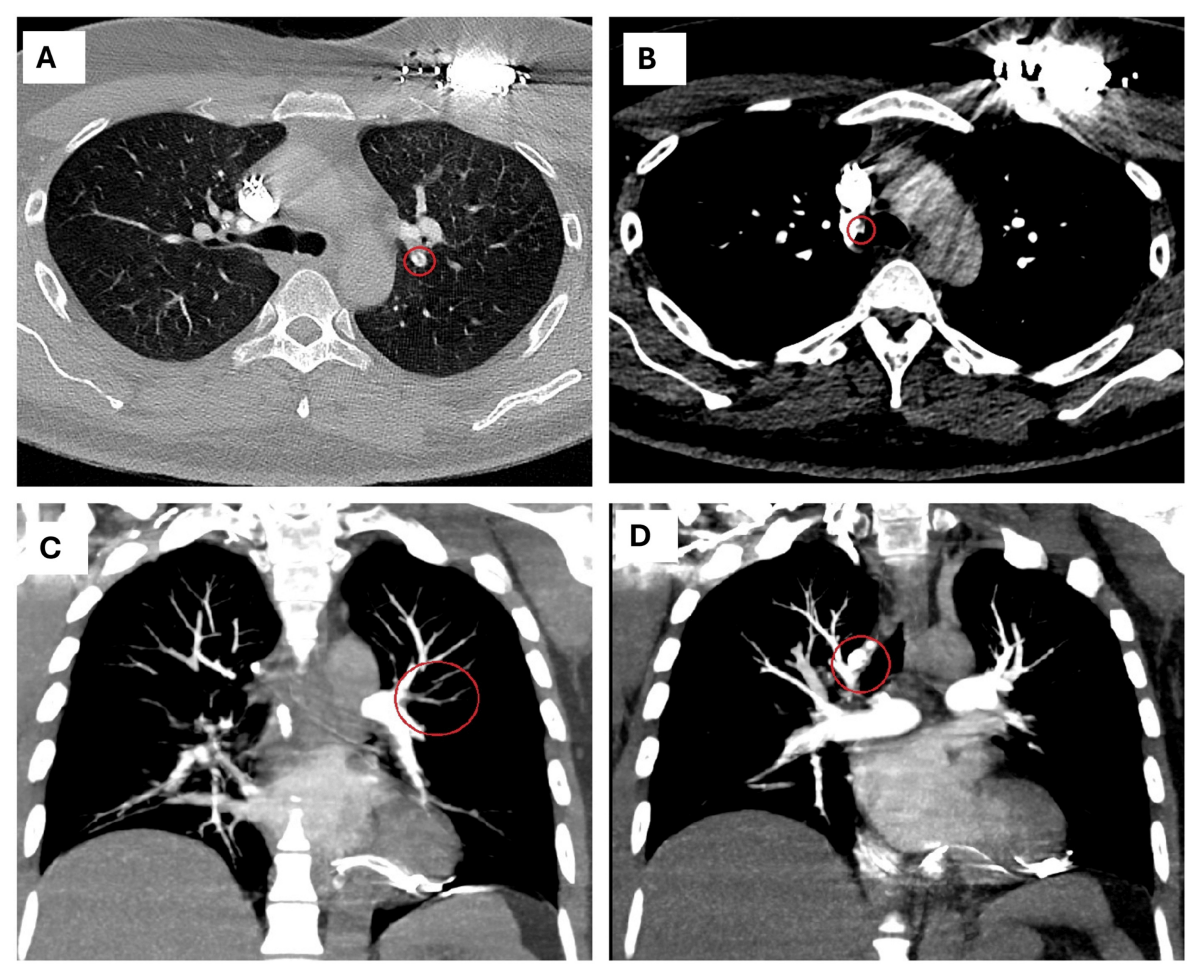
The patient’s computed tomography-pulmonary embolism (CT-PE) protocol scan A) Axial view demonstrating the left upper lobe segmental artery filling defect, encircled in red; B) Axial view demonstrating the right lower lobe segmental artery filling defect, encircled in red; C) Coronal view demonstrating the left upper lobe segmental artery filling defect, encircled in red; D) Coronal view demonstrating the right lower lobe segmental artery filling defect, encircled in red.

A d-dimer was requested due to presyncopal and anginal symptoms and returned abnormal at 0.643 milligrams per liter (mg/L) (reference value: <0.5 mg/L). Given his symptomatic state, he underwent emergent placement of a temporary transvenous pacemaker (TTVP) followed by an urgent computed tomography scan - PE protocol, which revealed bilateral pulmonary emboli (Figures 2a-2d).

He was immediately initiated on therapeutic subcutaneous twice daily low-molecular-weight heparin, enoxaparin 80 milligrams [8].

**Figure 3:**
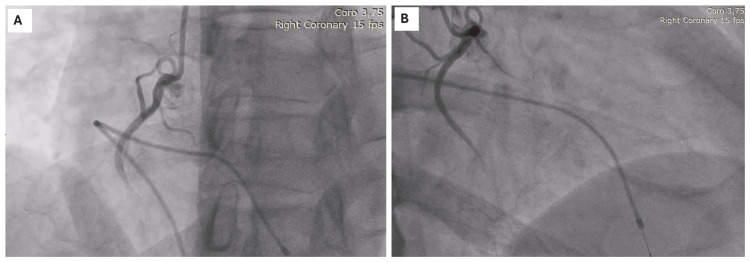
The patient’s transradial coronary angiography (TR-CAG) A) Left anterior oblique (LAO) view of the right coronary artery (RCA) demonstrates the coronary slow flow (CSF) phenomenon, very mild luminal irregularities, and a temporary transvenous pacemaker (TTVP); B) Right anterior oblique (RAO) view of the RCA demonstrates the CSF phenomenon, very mild luminal irregularities, and TTVP.

The patient was subsequently admitted to the cardiac care unit, where he underwent further diagnostic testing. Routine investigations, including complete blood count, renal function tests, liver function tests, and thyroid cascade, were normal. His high-sensitivity cardiac troponin I and NT-pro-brain natriuretic peptide were also normal. A bedside 2-dimensional transthoracic echocardiogram (2D-TTE) was performed and revealed a preserved left ventricular ejection fraction of 50-55% with mild inferolateral hypokinesis (tricuspid annular systolic plane excursion (TAPSE) of 14 millimeters; reference: ≥ 17 millimeters) and mild pulmonary hypertension (right ventricular systolic pressure (RVSP) of 40-45 mmHg; reference range: 15-30 mmHg) [13,14]. In light of his symptoms and abnormal ECG and 2D-TTE, the patient proceeded to transradial coronary angiography, which revealed very mild luminal irregularities with Thrombolysis in Myocardial Infarction II (TIMI II) slow flow (CSF) in the right coronary artery (Figure 3) [15].

Other typical etiologies, such as Lyme disease, sarcoidosis, myocarditis, or electrolyte abnormalities, were considered; however were excluded by lack of endemicity, normal serum angiotensin-converting enzyme (ACE) levels and several, complete electrolyte panels. A cardiac magnetic resonance imaging (cMRI) scan to assess for myocarditis was precluded due to the TTVP. Despite a five-day window for spontaneous resolution, a dual-chamber pacemaker (DCPM) was eventually implanted to prevent a healthcare-associated infection, such as endocarditis, from developing due to protracted exposure to the temporary pacing wire [1].

During his ensuing hospitalization, the patient also underwent exhaustive rheumatologic, immunologic, tumor marker, and thrombophilia panel testing; however, these all returned unremarkable, considering he was fully anticoagulated. His tentative diagnosis at this juncture was presumed to be PE-associated advanced heart block. In the interim, he was counseled extensively with respect to smoking cessation and commenced on aspirin, high-intensity statin and combined angiotensin receptor blocker-calcium channel blocker for his borderline hypertension and CSF [16,17]. He was subsequently de-escalated to the general cardiology ward for continued observation for one day post-procedure and then discharged with scheduled outpatient Cardiology and Pulmonology follow-up appointments. After one month of follow-up, the patient’s d-dimer was within normal limits on GDMT, including DOAC with aspirin, rosuvastatin, valsartan-amlodipine, pantoprazole, and apixaban. Interrogation of his DCPM revealed minimal interval usage, with Electrophysiology plans for eventual explantation, pending normalization of the cardiac conduction.

## Discussion

3°AVB, also known as CHB, signifies a critical disruption within the cardiac conduction system [18,19]. Normally, atrial electrical impulses traverse the atrioventricular node (AVN) towards the ventricles [20]. This interruption culminates in complete atrioventricular (AV) dissociation, in which each chamber contracts independently at its intrinsic rate. As a result, the atrial rate, dictated by the sinoatrial (SA) node, becomes markedly more rapid than the ventricular rate, the latter of which is established by a slower escape rhythm originating within the ventricles or the dysfunctional AV node [2]. This marked dissociation has deleterious sequelae, such as heart failure and death, necessitating emergent diagnosis and intervention [20]. This condition is commonly caused by fibrosis and sclerosis of the conduction system, ischemic heart disease, increased vagal tone, and certain medications, including digitalis, calcium channel blockers, beta-blockers, quinine, and amiodarone [1].To the authors' knowledge, there is a paucity of literature describing this potential association with PE [21,22].

PE is the third most common cause of cardiovascular death, following ACS and CVEs. Initially recognized by Virchow in the 19th century, PE can manifest with a constellation of characteristic ECG findings. These include sinus tachycardia, RBBB, ST-T wave abnormalities in the pertinent precordial leads, and the classic S1Q3T3 pattern [23,24]. The prevalence of RBBB in PE cases ranges from 6% to 69%, attributed to the RBBB's superficial location on the right ventricular side of the interventricular septum, which makes it susceptible to right ventricular strain [25].

A possible pathophysiological basis for CHB in the setting of PE centers on a pre-existing left bundle branch block (LBBB). This theory postulates that the hemodynamic stress imposed by PE can induce a conduction abnormality in a previously compromised conduction system [8]. In patients with LBBB, the transmission is delayed, forcing the impulse to detour through the less efficient Purkinje fibers. As PE can further accentuate right ventricular strain, it may transiently compromise RBB conduction, potentially leading to CHB. Notably, PE-associated CHB is more frequently observed in patients with pre-existing LBBB, suggesting a conduction vulnerability [17]. Furthermore, PE-induced hypotension, bradycardia, and coronary artery dilation can activate the Bezold-Jarisch reflex, a parasympathetic response that might potentiate pre-existing AV block within a compromised conduction system [26].

CSF, described by Tambe et al. in 1972, manifests with sluggish coronary artery perfusion in the absence of obstructive epicardial coronary artery disease [27]. This phenomenon is identified in approximately 1% of patients undergoing coronary angiography and up to 4% of individuals experiencing ACS. Studies have identified a potential demographic predilection, with a higher prevalence in males than in females. Additionally, conventional cardiovascular risk factors such as hypertension, dyslipidemia, tobacco use, metabolic syndrome, and obesity appear to play a crucial maladaptive role in the CSF milieu [28]. The most commonly involved vessel is the left anterior descending artery (50-90%), followed by the right coronary artery (28-45%), and the left circumflex artery (20%) [29]. Patients typically present with unstable angina, non-ST-segment elevation acute coronary syndrome, and ST-segment elevation acute coronary syndrome.

In this case, the patient's male gender and prominent smoking history are established risk factors for CSF, selectively affecting the right coronary artery [24]. We do acknowledge other possibilities of PE, such as cancer-associated thrombosis or inherited or acquired thrombophilia, despite the pitfalls of testing during this period. We attributed both PE and CSF due to significant tobacco use; however, it was virtually impossible to differentiate which was the preceding index condition. While a direct causal link between PE and CSF remains elusive, the authors postulate that acute PE can accentuate right ventricular strain, imposing a hemodynamic burden that may compromise coronary blood flow, especially during diastole [26] (Table 1).

**Table 1:** Summary of the reported cases with pulmonary embolism-associated advanced heart block

Study	Year	Age (Years)	Sex (Male, Female)	Type of Pulmonary embolism (PE)	Type of complete heart block (CHB)	Management	Outcome
Elias et al. [9]	2004	68 years	Male	Massive PE (right pulmonary artery trunk to lobar and segmental branches)	Persistent CHB	Thrombolysis with streptokinase and temporary pacing	Died after an attempted mechanical extraction of thrombi
Zuin & Rigatelli [21]	2017	68 years	Female	Massive PE (right main and left lobar)	Transient CHB	Systemic thrombolysis with recombinant tissue plasminogen activator and temporary pacing	Full recovery of atrioventricular (AV) conduction, pacing was not required long-term
Olson et al. [22]	2019	78 years	Male	Bilateral PE (right interlobar and upper lobar, left upper lobar and left lower lobar)	1^st ^degree atrioventricular (AV) block with new right bundle branch block (RBBB) and pre-existing left bundle branch block (LBBB) → Intermittent CHB	Systemic thrombolysis with tissue plasminogen activator and temporary pacing	Recovery to 1^st ^degree AV block, Dual chamber permanent pacemaker implanted
Ismail et al. [10]	2021	52 years	Male	Saddle PE	Bifascicular block (RBBB and left anterior fascicular block (LAFB)) → intermittent CHB	Heparin and temporary pacing	Recovery, pacing discontinued
Ma et al. [12]	2023	54 years	Male	Massive PE (right upper lobe, right lower lobe and left lower lobe)	Intermittent CHB	Anticoagulation	Recovered, pacing not required
Kim et al. [11]	2024	91 years	Female	Bilateral PE (distal left main and both lobar and segmental arteries)	Persistent CHB	Catheter-directed thrombolysis with alteplase and temporary pacing	Full recovery, no pacemaker needed
Present Case	2025	35 years	Male	Bilateral segmental PE (right lower and left upper lobe)	Persistent CHB	Anticoagulation, guideline-directed medical therapy (GDMT) for coronary slow flow (CSF), Permanent dual-chamber pacemaker	Recovery, pacemaker with minimal pacing burden

Consequently, the impaired diastolic perfusion could theoretically contribute to the development of CSF, particularly within the right coronary artery, which perfuses a significant portion of the right ventricular myocardium and the AV node [30]. Thus, acute PEs may have led to CSF in this vessel, thereby compromising AV node perfusion and ultimately triggering CHB.

## Conclusions

This case report describes a rare, coincident presentation of suspected PE-associated advanced heart block. While CHB and PE are individually well-characterized entities, their concomitant presentation warrants further investigation into the underlying pathophysiology, possibly implicating CSF. The patient was successfully managed with GDMT for PE and underwent DCPM implantation for the CHB. The clinician should be cognizant of atypical etiologies such as PE in young adults presenting with advanced heart block.

## References

[1] Kusumoto FM, Schoenfeld MH, Barrett C (2019). 2018 ACC/AHA/HRS guideline on the evaluation and management of patients with bradycardia and cardiac conduction delay: a report of the American College of Cardiology/American Heart Association Task Force on clinical practice guidelines and the Heart Rhythm Society. Circulation.

[2] Glikson M, Nielsen JC, Kronborg MB (2021). 2021 ESC Guidelines on cardiac pacing and cardiac resynchronization therapy: developed by the task force on cardiac pacing and cardiac resynchronization therapy of the European Society of Cardiology (ESC) with the special contribution of the European Heart Rhythm Association (EHRA). Eur Heart J.

[3] Brockman SK, Manlove A (1965). Cardiodynamics of complete heart block. Am J Cardiol.

[4] Rowe JC, White PD (1958). Complete heart block: a follow-up study. Ann Intern Med.

[5] Konstantinides SV, Barco S, Lankeit M, Meyer G (2016). Management of pulmonary embolism: an update. J Am Coll Cardiol.

[6] Barco S, Valerio L, Gallo A (2021). Global reporting of pulmonary embolism-related deaths in the World Health Organization mortality database: vital registration data from 123 countries. Res Pract Thromb Haemost.

[7] Duffett L, Castellucci LA, Forgie MA (2020). Pulmonary embolism: update on management and controversies. BMJ.

[8] Stevens SM, Woller SC, Baumann Kreuziger L (2021). Executive Summary: antithrombotic therapy for VTE disease: second update of the CHEST guideline and expert panel report. Chest.

[9] Elias J, Kuniyoshi R, Moulin B (2004). Syncope and complete atrioventricular block related to pulmonary thromboembolism. Arq Bras Cardiol.

[10] Ismail Z, Salabei JK, Stanger G, Asnake ZT, Frimer L, Smock A (2021). Third-degree heart block associated with saddle pulmonary embolism: a rare sequelae of COVID-19-induced hypercoagulable state. Cureus.

[11] Kim M, Seo CO, Kim H, Kim HR, Kim K, Kang MG, Park JR (2024). Case report: complete atrioventricular block in an elderly patient with acute pulmonary embolism. Front Cardiovasc Med.

[12] Ma M, Liang S, He Y, Wang H (2023). Case report: the presence of third-degree atrioventricular block caused by pulmonary embolism masquerading as acute ST-segment elevation myocardial infarction. Front Cardiovasc Med.

[13] López-Candales A, Edelman K, Candales MD (2010). Right ventricular apical contractility in acute pulmonary embolism: the McConnell sign revisited. Echocardiography.

[14] McConnell MV, Solomon SD, Rayan ME, Come PC, Goldhaber SZ, Lee RT (1996). Regional right ventricular dysfunction detected by echocardiography in acute pulmonary embolism. Am J Cardiol.

[15] Gibson CM, Cannon CP, Daley WL (1996). TIMI frame count: a quantitative method of assessing coronary artery flow. Circulation.

[16] Aparicio A, Cuevas J, Morís C, Martín M (2022). Slow coronary blood flow: pathogenesis and clinical implications. Eur Cardiol.

[17] Wang X, Nie SP (2011). The coronary slow flow phenomenon: characteristics, mechanisms and implications. Cardiovasc Diagn Ther.

[18] Knabben V, Chhabra L, Slane M (2023). Third-degree atrioventricular block. StatPearls [Internet].

[19] Hashim AT, Mehmood Q, Ahmad S (2023). Complete heart block. Clinical and Surgical Aspects of Congenital Heart Diseases.

[20] Power DA, Lampert J, Camaj A (2022). Cardiovascular complications of interatrial conduction block: JACC state-of-the-art review. J Am Coll Cardiol.

[21] Zuin M, Rigatelli G (2017). Complete heart block as presenting symptom of massive pulmonary embolism in an elderly patient. J Geriatr Cardiol.

[22] Olson PC, Cinelli M, Kurtovic E, Barsoum E, Spagnola J, Lafferty J (2020). Complete atrioventricular block caused by pulmonary embolism: a case report and review of literature. Heart Lung.

[23] Piazza G (2020). Advanced management of intermediate- and high-risk pulmonary embolism: JACC focus seminar. J Am Coll Cardiol.

[24] Pastori D, Cormaci VM, Marucci S (2023). A comprehensive review of risk factors for venous thromboembolism: from epidemiology to pathophysiology. Int J Mol Sci.

[25] Frisbie JH, Sharma GV (2009). Right bundle branch block as a screening test for pulmonary embolism in chronic spinal cord injury. Arch Phys Med Rehabil.

[26] Lim GB (2024). Explaining how a cardiac reflex causes syncope. Nat Rev Cardiol.

[27] Tambe AA, Demany MA, Zimmerman HA, Mascarenhas E (1972). Angina pectoris and slow flow velocity of dye in coronary arteries-a new angiographic finding. Am Heart J.

[28] Chalikias G, Tziakas D (2021). Slow coronary flow: pathophysiology, clinical implications, and therapeutic management. Angiology.

[29] Alvarez C, Siu H (2018). Coronary slow-flow phenomenon as an underrecognized and treatable source of chest pain: case series and literature review. J Investig Med High Impact Case Rep.

[30] Yamamoto T (2018). Management of patients with high-risk pulmonary embolism: a narrative review. J Intensive Care.

